# Supplementary figures and images for: Anti-inflammatory role of APRIL by modulating regulatory B cells in antigen-induced arthritis
Source: PLoS One. 2024 May 1;19(5):e0292028. doi: 10.1371/journal.pone.0292028 (PMC11062543; doi:10.1371/journal.pone.0292028)

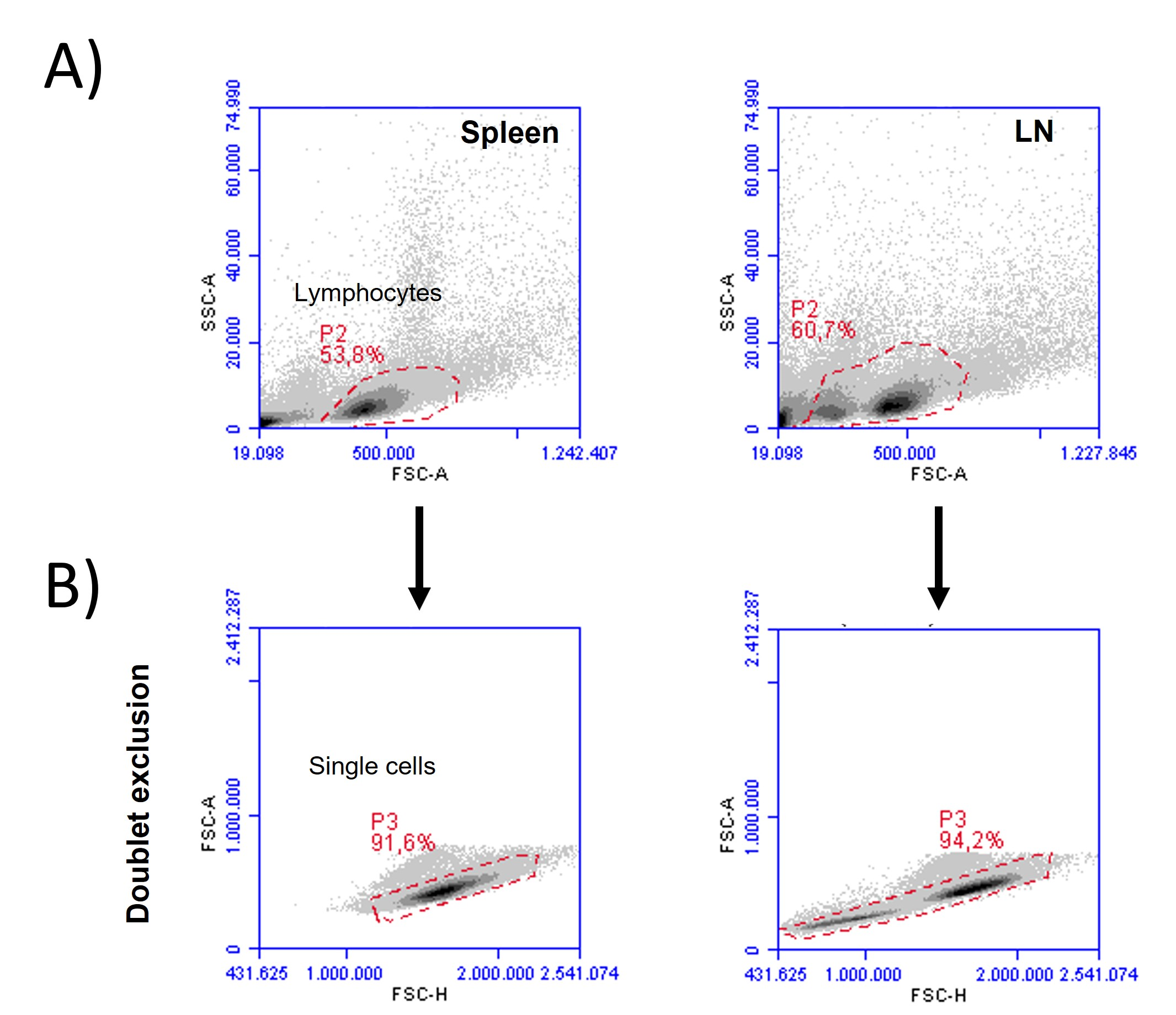

Supplement: S1 Fig — A, Spleen, and lymph node cells, obtained from littermate control mice as described in Material and Methods, were acquired on a BD Accuri C6 cytometer (BD Bioscience, USA). Dot plots representing the gate strategy for the lymphocyte total population region in the FSC (size) and SSC (complexity) parameters. B, Dot plot representing the gate strategy in the region referring to individual cells (singlets), excluding cells aggregated into doublets, combining the parameters of FSC-A (forward scatter area) x FSC-H (forward scatter height). After applying the initial gate strategies depicted, lymphocyte subsets were analyzed using the program Cflow (BD Bioscience, USA) as following, CD19+CD5+ B1 cells as referred in [35]; CD19+CD21hiCD23hiCD24+T2-MZP B regs and CD4+CD25+FOXP3+ T regs as referred in [36]. (TIF) [file pone.0292028.s001.tif]
