# Supplementary material for: Anti-inflammatory role of APRIL by modulating regulatory B cells in antigen-induced arthritis
Source: PLoS One. 2024 May 1;19(5):e0292028. doi: 10.1371/journal.pone.0292028 (PMC11062543; doi:10.1371/journal.pone.0292028)
Supplement: Attachment — Submitted filename: Manuscript_review.pdf [file pone.0292028.s002.pdf]

The manuscript titled "Anti-inflammatory role of APRIL by modulating regulatory B cells in antigen-induced arthritis" explores APRIL's role in regulating the B cell-mediated inflammatory response in an antigen-induced arthritis mouse model. The study utilizes an mBSA-induced arthritis model, uncovering that APRIL overexpression correlates with reduced joint swelling, disease scores, and inflammatory infiltration in knee joint tissues. The study is focused on APRIL determined behavior of immune cells (mostly B cells) in mouse arthritis model.

While the anti-inflammatory role of APRIL has been documented in other contexts, its function under specific inflammatory conditions, such as arthritis, necessitates further examination. This study provides new insights into APRIL's anti-inflammatory effects in a mouse arthritis model. However, before publication, improvements are required. The following are my major and minor concerns:

Major Concerns:

- The study demonstrates differences in specific B cell and CD4+ T cell subpopulations, yet it remains unclear whether the total number of various immune cell types (T cells (CD4+, CD8+), B cells, myeloid cells (neutrophils, macrophages, dendritic cells, etc.)) is impacted by APRIL overexpression. A comparison of these numbers in the joint area, lymph nodes, and spleen is necessary
- All flow cytometry data should include a complete gating strategy. If necessary, part of this data can be placed in supplementary material to avoid overloading the figures
- In the histopathological comparison, was morphometry employed? How were conclusions regarding the inflammatory infiltrate drawn? Were there observable differences in other morphological changes between control and APRIL-tg groups? If so, these should be presented with corresponding morphometry
- The discrepancy in IgG titer is mostly attributed to the IgG1 subclass (0.974 (APRIL-Tg mice) vs 0.594 (ctrl mice)). However, the actual IgG1 titer shows a less significant difference (0.666 (APRIL-Tg mice) vs 0.516 (ctrl mice). How do authors explain this? The reasons for this and the titers of other subclasses should be addressed
- The study's conclusion lacks clarity regarding the precise role of APRIL-driven mechanisms linked to B cell behavior in arthritis. It is essential to elucidate why B cells, in particular, are the focus over other cell types in the progression of arthritis in the context of APRIL's effects (in fact APRIL affects many cell types). Additionally, the rationale for emphasizing B cells should be made explicit, considering their role in comparison to other immune cells within the APRIL-mediated response in arthritis

Minor Concerns:

- All plots should demonstrate individual points
- In Figure 2, cellularity in lymph nodes and spleen is compared, but the methodology is unclear. If flow cytometry was used, how was it normalized? This information is needed
- The study should address whether there is a gender dependency in APRIL expression and justify the exclusive use of male mice

- Details on single-cell suspension preparation should be added to the "Materials and Methods" section
- The image (histological slides) acquisition technique needs description and inclusion in the "Materials and Methods" section (microscope, magnification etc.)
- The current total of 11 figures could be consolidated into 5-6 for ease of reading, combining figures where feasible
- Figure 3 requires redesigning for clarity, particularly in labeling images A and D, which currently reference healthy mice but appear to indicate acute and chronic arthritis states in the figure
